# 18-month results of double-fanged 5-0 polypropylene suture transscleral bag fixation in subluxated cataracts

**DOI:** 10.5935/0004-2749.20230022

**Published:** 2022-02-18

**Authors:** Sergio Canabrava, Gustavo Rodrigues, Natan Namem Halabi, Ana Clara Rezende, Mayara Cardoso

**Affiliations:** 1 Cataract Department, Santa Casa de Misericórdia de Belo Horizonte, Belo Horizonte, MG, Brazil

**Keywords:** Cataract, Phacoemulsification, Lense; intraocular, Suture technique, Visual acuity, Catarata, Facoemulsificação, Lente intraocular, Técnica de sutura, Acuidade visual

## Abstract

**Purpose:**

To evaluate the stability and efficacy of the double-flanged 5-0 polypropylene suture to fixate subluxated cataracts at 18 months and the possible complications of this new technique

**Methods:**

This technique uses a 5-0 polypropylene monofilament to create two flanges with a thermocautery, for fixation of a capsular tension segment to the sclera to fix the subluxated capsular bag. This technique was implemented in 17 eyes requiring intraocular lens implantation in a setting of zonular dialysis due to trauma, Marfan syndrome, microspherophakia, idiopathic disease, and post-phacoemulsification status.

**Results:**

Follow-up of the patients occurred at 18 months. Best-corrected visual acuity improved significantly from 0.85 to 0.39 (logMAR), whereas the spherical and cylindrical refractive errors and intraocular pressure remained stable from preoperation. No suture photodegradation or pseudophacodonesis were detected.

**Conclusion:**

The double-flanged 5-0 polypropylene suture transscleral bag fixation technique has shown favorable long-term outcomes in terms of bag intraocular lens/complex fixation and stability. In eyes with zonular weakness or dialysis, this technique appears to be a safe and knotless option for cataract surgery.

## Abstract (pt)

**Objetivo:**

Avaliar a estabilidade e eficácia da técnica *double-flanged* com sutura de 5-0 polipropileno para fixação de cataratas subluxadas aos 18 meses e as possíveis complicações desta nova técnica.

**Métodos:**

Esta técnica utiliza um monofilamento de polipropileno 5-0 para criar dois flanges com um termocautério para fixar um Segmento de Tensão Capsular na esclera a fim de estabilizar o saco capsular subluxado. Esta técnica foi implementada em 17 olhos que necessitavam do implante de lente intraocular em casos de diálise zonular devido a trauma, síndrome de Marfan, microesferofacia, subluxação idiopática ou pós-facoemulsificação que provocou subulxação do saco capsular intraoperatória.

**Resultados:**

O seguimento dos pacientes foi de 18 meses. A acuidade visual corrigida melhorou significativamente de 0,85 para 0,39 (logMAR), enquanto os erros de refração esféricos e cilíndricos e a pressão intraocular permaneceram estáveis. Nenhuma fotodegradação de sutura ou pseudofacodonese foi encontrada.

**Conclusão:**

A técnica *double-flanged* para fixação transescleral de saco capsular com sutura de 5-0 polipropileno mostrou resultados de estabilidade de longo prazo para o complexo lente/saco capsular. Então, aparenta ser uma opção segura para cirurgia de catarata, sem necessidade pontos, em olhos com fraqueza zonular ou diálise.

## INTRODUCTION

Surgical treatment of subluxated cataracts is a challenging task, even for experienced anterior segment surgeons. The choice of technique and devices depends on the degree of zonulopathy and the pathophysiologic origin of the zonular abnormality^(1)^.

In cases in which the zonular dialysis is less than 120°, a standard capsular tension ring (CTR) is commonly used. However, in cases with more extensive zonular laxity and progressive pathologies such as pseudoexfoliation, Marfan syndrome, Weill–Marchesani syndrome, and deficiency of sulfite oxidase, in which subluxation can worsen with time, fixation of the capsular bag in the sclera with a modified CTR or a capsular tension segment (CTS) is recommended^(1,2^, ^3,4)^.

Although scleral fixation is a well-known and reproducible technique, it requires a high degree of surgical skill and expertise; it also increases surgical time. Furthermore, polypropylene 10-0 has been shown to hydrolyze within 5 to 10 years; thus, scleral fixations are subject to suture degradation, pseudophacodonesis, and late intraocular lens (IOL) decentration^(5,6^, ^7)^.

To overcome these challenges, we became interested in using a thicker monofilament combined with a flanged technique. In 2017, we described a knotless, double-flanged technique using a 5-0 polypropylene suture^(8)^ (Ethicon Johnson, USA). In 2020, we proposed the use of this modified technique for fixating the capsular bag in patients with ectopia lentis^(9)^.

Since we published the first report of double-flanged polypropylene suture in 2017^(8)^, many techniques have been reported using this new kind of suture, including how to fixate a single-piece IOL^(9)^, a foldable IOL, iris^(10)^, and artificial iris. However, the medium- to long-term stability of this technique has yet to be evaluated. Thus, in this paper, we present the 18-month data of our double-flanged transscleral bag fixation technique using a 5-0 polypropylene suture for fixing the CTS to the sclera in patients with zonular dialysis >120º.

## METHODS

This prospective case series is composed of 17 eyes of 17 patients with zonular dialysis >120° who required IOL implantation in the setting of zonular dialysis due to trauma (six eyes), Marfan syndrome (three eyes), microspherophakia (two eyes), idiopathic disease (two eyes), post-phacoemulsification status (four eyes), or eyes that have previously undergone cataract surgery and that evolved with IOL bag dislocation, thus requiring a second surgery for IOL implantation and bag centration (see Tables 1 and 2 for population characterization). In patients with zonular dialysis >180°, such as those with microspherophakia, we used two CTSs to fixate the capsular bag.

**Table 1:** Age and sex of the patients

Age range, y	Male	Female
30-39	0	2
40-49	0	0
50-59	0	2
60-69	5	5
70-79	3	0
Total	8	9

**Table 2:** Cause of zonular weakness of patients

Cause of zonular weakness	Number of patients
Trauma	4
Intraoperative phacoemulsification	7
Ectopia	2 (Marfan) 1 (Microspherophakia)
Uveitis	1
Idiopathic	1

The preoperative examination included best-corrected visual acuity (BCVA), detailed dilated pupil slit-lamp examination using a gonio lens to estimate disinsertion, and measurement of intraocular pressure (IOP), which was measured using the Goldmann applanation tonometer. Postoperative evaluation included suture breakage, extrusion flange, conjunctival and scleral erosion, BCVA recorded 18 months after surgery, IOP measured at 15 and 30 days, and spherical and cylindrical refractive errors obtained by autorefraction when eyes were not dilated, which were recorded 30 days and 18 months postoperatively to evaluate long-term stability.

To rule out systemic syndromes associated with ectopia lentis, a detailed systemic examination was also included as part of the preoperative examination.

All procedures were performed by the same surgeon (S.C.) between October 2017 and November 2019. Ethical committee approval for the study was obtained by the authors from Plataforma Brazil (No. CAAE 69382517.6.00005138 submitted May 11, 2017, Santa Casa de Belo Horizonte), and the study followed the tenets of the Declaration of Helsinki. All patients were asked to sign an informed consent form before treatment.

Measurement of visual acuity was recorded in Snellen and then converted to logMAR for statistical analysis. Follow-up was scheduled at 1, 3, 6, 12, and 18 months. We report the results at 18 months postoperatively.

We performed statistical analysis using a commercially available statistical software package (IBM Corp., released 2015, IBM SPSS Statistics for Windows, version 20.0, Armonk, NY). To compare BCVA values over time, a paired *t* test was computed. A p value <0.05 was considered statistically significant.

### Technique

We have previously described in detail the double-flanged polypropylene technique^(8)^. In summary, using a 26-gauge needle, we make a scleral tunnel in the same quadrant of the zonular weakness. A well-angulated tunnel can be created by making the tunnel as parallel as possible and about 2.0 mm from the limbus.

**Figure 1:**
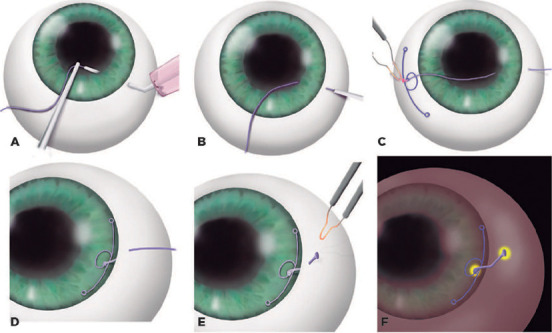
(A) 5-0 Prolene is inserted in the needle lumen. (B) Prolene is drawn out from the sclera. (C) The monoflament is heated and shaped into the first fange. (D) CTS is inside the bag. (E) The second fange is created. (F) The second fange is inserted inside the scleral tunnel.

**Figures 2:**
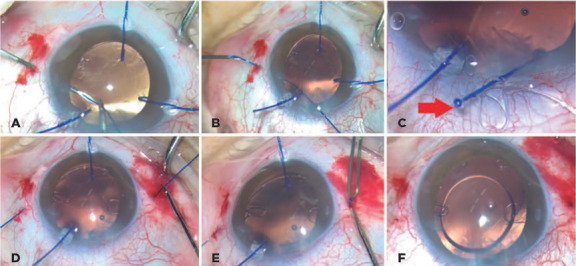
(A) 5-0 Prolene is inserted in the needle lumen. Notice that three iris polypropylene hooks support the bag. (B) Prolene is drawn out from the sclera using a 26-G needle. (C) The 5.0 polypropylene inside the CTS eyelet and the fst fange made. (D) CTS inside the bag. (E) The second fange is created. (F) The second fange is inserted inside the scleral tunnel.

When the needle is in the anterior chamber, we use 23-gauge micro-forceps through the corneal incision to place a 5-0 polypropylene monofilament into the 26-gauge inner cavity of the needle (Figures 1A and 2A). Next, the needle externally guides the suture end within the sclera (Figures 1B and 2B), and we pass an adjustable 5.0 polypropylene suture through the anterior chamber: one end out of the corneal incision and the other end out of the sclera.

**Figure 3:**
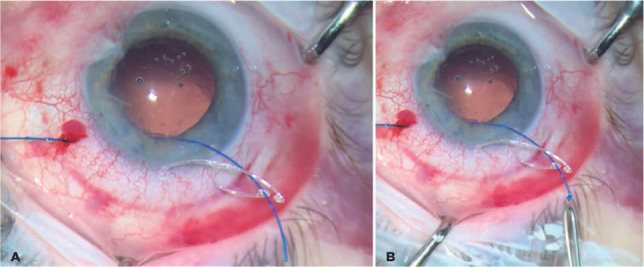
(A) 5-0 Prolene is seen inside the eyelet of the CTS. (B) The first fange is made.

The external cornea side of the 5-0 suture is placed in the suturing eyelet of the CTS (Figure 3A), heated, and shaped into a flange using a bipolar portable cautery outside the eye (Figures 1C, 2C, and 3B) to make the first flange. After this procedure, the CTS/5-0 monofilament complex is created. Subsequently, the CTS is placed in the same quadrant of the zonular weakness (Figures 1D and 2D).

Finally, the external scleral side of the monofilament is cut to approximately 2 mm to adjust the size and to place the CTS in a better position. The monofilament is then heated and shaped into a second flange using the same bipolar portable cautery (Figures 1E and 2E).

**Figure 4:**
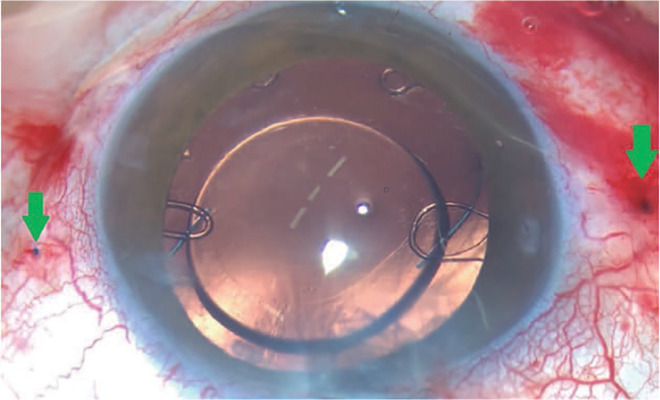
A case of microspherophakia with two CTSs fixated at the sclera using the double-fanged technique on both sides and the IOL implanted in the bag.

Next, this second flange is inserted into the scleral tunnel (Figures 1F and 2F) using McPherson forceps (Sklar, West Chester, PA, USA). The IOL is in the bag (Figure 4).

## RESULTS

There was a statistically significant difference in visual acuity between the preoperative and 18-month visits. Preoperatively, 12% of the eyes had a BCVA equal to or better than 20/80 Snellen; 18 months postoperatively, 78% had a BCVA better than or equal to 20/80 Snellen (Table 3). Overall, the BCVA improved significantly (p=0.001) from 0.85 (SD 0.28) to 0.39 (SD 0.41) (logMAR). The spherical error passed from 0.37 D (SD 0.88) at 30 days to 0.41 D (SD 0.85) at 18 months postoperatively. This is considered stable, as the paired *t* test showed no change (p=0.698). The cylindrical error remained stable from -1.57 D (SD 1.23) at 30 days postoperatively to -1.74 D (SD 1.29) at 18 months postoperatively. The paired *t* test showed no difference (p=0.069).

**Table 3:** Preoperative and 18-month postoperative BCVA

VA Snellen	VA logMAR	BCVA, preop			BCVA, 18 mo postop		
		Frequency	%	Cumulative (%)	Frequency	%	Cumulative (%)
**20/20**	0	0	0	**0**	3	18	**18**
**20/25**	0.1	0	0	**0**	3	18	**35**
**20/32**	0.2	1	6	**6**	3	18	**53**
**20/40**	0.3	0	0	**6**	1	6	**59**
**20/50**	0.4	0	0	**6**	1	6	**65**
**20/63**	0.5	1	6	**12**	1	6	**71**
**20/80**	0.6	0	0	**12**	1	6	**76**
**20/100**	0.7	6	35	**47**	2	12	**88**
**20/160**	0.9	1	6	**53**	0	0	**88**
**20/200**	1	6	35	**88**	0	0	**88**
**20/400**	1.3	2	12	**100**	2	12	**100**
**Total**		**17**	**100**		**17**	**100**	

The IOP measured preoperatively was 14.2 mmHg (SD 4.49) and increased significantly at 15 days to 18.9 mmHg (SD 8.76) but then stabilized at 15 mmHg (SD 7.94) at 30 days postoperatively. This can be considered stable over time (p=0.080).

Table 4 recaps all results.

**Table 4:** Recap results preoperatively and at 15 days, 30 days, and 18 months postoperatively

	Preop	15 days	30 days	18 mo
Mean BCVA	0.85 logMAR			0.39 logMAR
Mean sphere			0.37 D	0.41 D
Mean cylinder			-1.57 D	-1.74 D
Mean IOP	14.18	18.94	15.00	

**Figure 5:**
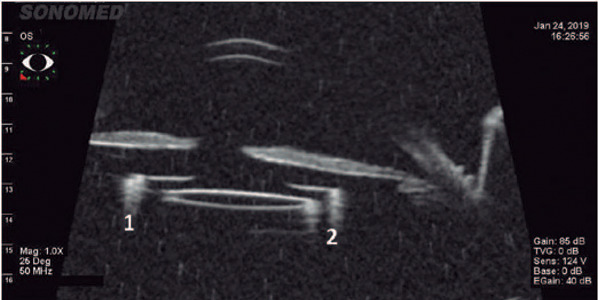
Ultrasound biomicroscopy (UBM) image showing the IOL in the right position. The numbers 1 and 2 show the capsular tension segments in a patient with microspherophakia.

We observed no scleral degradation or polypropylene suture degradation. To observe the complex CTS-IOL positions inside the eye, we performed ultrasound biomicroscopy in all eyes at 18 months postoperatively using the UBM Plus (Accutome, Keeler and Microsurgical Technology, USA). The ultrasound biomicroscopy images (Figure 5) showed that the 5-0 polypropylene suture did not touch the ciliary body.

We recorded some complications, including blebitis and glaucoma in one case, tractional retinal detachment after 12 months in one case, and contraction capsular syndrome in one case. In one case, one flange touched the posterior section of the iris, as the scleral tunnel was made less than 2 mm from the limbus. This case did not experience any clinical complications.

**Figure 6:**
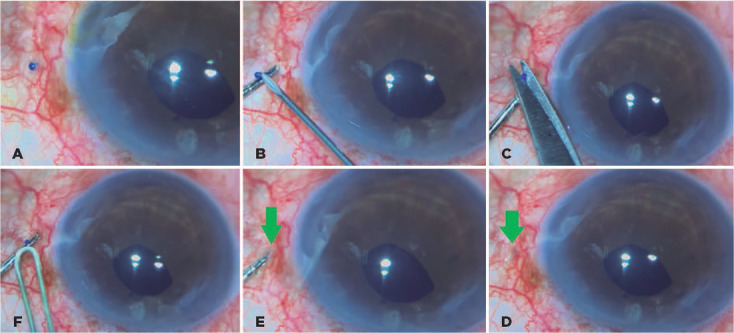
(A) Flange end extrusion. (B) Externalization of the polypropylene segment using a 26-G needle. (C) The end of the fange is cut. (D) A 1-mm-length fange. (E) Inserting the end of the fange in the scleral tunnel. (F) The fange is buried in the scleral tunnel.

There was one case of flanged extrusion in the long external polypropylene suture. Thus, it was necessary to return the patient to the operating room and reduce the flange and length of the external polypropylene (Figure 6). In addition, although in our previous four-flanged scleral fixation study^(9)^ we found patients with conjunctival erosion, we did not encounter any conjunctival or scleral erosion in this case series. One case had a limited vitreous hemorrhage while creating the scleral tunnel. Of 17 eyes, 15 IOLs were stable at 18 months and two were slightly decentered. No uveitis was observed.

## DISCUSSION

The management of congenitally dislocated cataracts in such syndromic cases has traditionally consisted of intracapsular cataract extraction and pars plana vitrectomy/lensectomy, but the large incision required for intracapsular cataract extraction induces significant postoperative corneal edema and astigmatism, and lensectomy often results in the requirement of aphakia glasses or aphakia contact lenses^(11)^.

If the subluxation is less than 120°, a conventional CTR is usually satisfactory for managing weakness. Instead, when the eyes present a weakness greater than 120° or zonular dialysis, centering and fixing the bag to the sclera may be required for satisfactory long-term stability^(12,13)^. Since Hara et al first presented the standard CTR^(14)^ in 1991, several authors have proposed different strategies that adapt the CTR to treat zonular dialysis^(15,16,17)^.

Traditionally, these solutions use a polypropylene 10-0 suture to anchor the bag to the sclera. Polytetrafluoroethylene (Gore-Tex) is used for the same purpose because of its longer lifetime^(18)^. However, the published literature has shown that 10-0 polypropylene sutures may degrade over time and, in the long term, result in dislocation of the IOL^(13,14,15)^.

To replace the standard 10-0 monofilament, we chose a 5-0 polypropylene monofilament because it is thicker^(19)^ and thus shows greater resistance to degradation and may reduce the risk of suture rupture. In addition, the 5-0 polypropylene monofilament is placed in the 26-gauge lumen and the needle guides the monofilament externally, which avoids the time-consuming and difficult maneuvers needed in the traditional approach to pass long needles in the anterior chamber using 10-0 polypropylene or 7-0 polytetrafluoroethylene sutures.

With regard to the technique, we used our double-flanged technique, inspired by Yamane’s flanged technique, which demonstrated both safety and stability in IOL secondary implants^(20)^.

Our first experience coupling this technique and the thicker monofilament was described in our 2017 study^(8)^. We continued using this technique for years, and its versatility was confirmed in 2020 when our group described in several studies the use of a 5-0 polypropylene to fixate a single-piece PMMA IOL in the sclera^(9)^. Other ophthalmology uses of a thicker monofilament have been reported by Kusaka^(21)^, who used a 6-0 monofilament to fixate iridodialysis, and by our group when we used a four-flanged technique to fixate a four-eyelets IOL to the sclera^(22)^.

Also important is the shape of the tunnel and the length of the external segment of the polypropylene. The surgeon needs to create a long and well-angulated tunnel, because if the needle is inserted directly into the sclera, the tunnel will be short, and insertion of the second flange inside the tunnel will thus be more difficult. The length of the external segment of the polypropylene needs to be approximately 2 mm; if the surgeon shortens the external end of the suture, the capsular bag can be tight. On the other hand, if the external polypropylene is too long, extrusion of the flange can occur, which occurred in one patient, and we had to intervene surgically to reduce the external length of the suture.

We confirmed that at 18 months after surgery, the flange bag-CTS complex weas stable and there was no scleral degradation in any of the 17 eyes with subluxated IOL that underwent surgery with this technique.

We recorded a few complications, including blebitis and glaucoma in one case. One patient previously had glaucoma, which was manageable with drops, and had a bag disinsertion during a previous phacoemulsification and then we proceeded with the double-flanged technique. In addition, the IOP increased, and it was necessary to perform trabeculectomy. Blebitis occurred 30 days later.

Other complications include tractional retinal detachment after 12 months in another case and contraction capsular syndrome in one case. Infection did not occur in any of the cases.The BCVA increased significantly over 18 months: a gain of 0.49 logMAR, which was superior to the 0.13 logMAR gain of the Yamane^(20)^ study at 24 months, despite the difference in preoperative values and the fact that the different techniques used made it difficult to establish a significant comparison. In 2011, Chee^(23)^ reported a noncomparative case series using a CTS with a 10.0 propylene suture: of 41 eyes, 22% had at least 20/40 Snellen preoperatively as compared with 94% at 12 months postoperatively, but it must be taken into account that much fewer eyes (only 18 of 41) were recorded at that follow-up. In our study, only 6% had a preoperative BCVA better than 20/40, but this figure was 59% at 18 months; all eyes examined preoperatively were recorded postoperatively.

Five patients had a postoperative BCVA that was worse than 20/80: three patients previously had ocular trauma and multiple procedures (retinal detachment, choroid thickening, epiretinal membrane), one patient previously had idiopathic uveitis, and one had multiple procedures after previous ocular trauma. The specific cause of the limited gain in BCVA after surgery was not determined. However, the refractive and IOP parameters remained stable.

The long-term results of the double-flanged 5-0 polypropylene transscleral bag fixation technique were comparable with those of traditional suture-held CTSs but does not require flaps, sutures, or glue. Equally, this technique is less traumatic for the ocular tissue, it is performed much faster, and it is easier to learn. Moreover, because this method uses a thicker monofilament and may prevent suture degradation, it may also avoid traditional complications of IOL misalignment or dislocation over time.

The greatest limitation of this study is the small number of patients operated on using this technique. Although we attained a follow-up period of at least 18 months, a larger number of eyes are preferable to achieve more conclusive results. A larger sample size also means that other complications can be identified. Furthermore, because of the specific nature of this technique, most eyes examined in this study were from patients between 60 and 69 years of age. Ideally, a more balanced population is preferred.

Overall, the double-flanged 5-0 polypropylene suture transscleral bag fixation technique has shown satisfactory long-term outcomes for IOL fixation and stability, and it appears to be a safe, knotless option for cataract surgery in eyes with zonular weakness or dialysis. However, to establish the exact outcomes with zonular weakness of different etiologies, further studies that include a larger number of patients and longer follow-up times are needed.
